# A randomized study of local anesthesia for pain control during intra-articular corticosteroid injection in children with arthritis

**DOI:** 10.1186/s12969-015-0034-8

**Published:** 2015-08-27

**Authors:** Jennifer E. Weiss, Kathleen A. Haines, Elizabeth C. Chalom, Suzanne C. Li, Gary A. Walco, Themba L. Nyirenda, Barbara Edelheit, Yukiko Kimura

**Affiliations:** Department of Pediatrics, Section of Rheumatology, Joseph M. Sanzari Children’s Hospital, Hackensack University Medical Center, 30 Prospect Ave., Hackensack, NJ 07610 USA; Saint Barnabas Medical Center, Pediatric Rheumatology, Livingston, NJ 07039 USA; Department of Anesthesiology & Pain Medicine, Seattle Children’s Hospital, University of Washington School of Medicine, 4800 Sand Point Way NE, Seattle, WA 98105 USA; Department of Research, Hackensack University Medical Center, Hackensack, NJ 07601 USA; Connecticut Children’s Medical Center, Pediatric Rheumatology, Hartford, CT 06106 USA

**Keywords:** Juvenile idiopathic arthritis, Intra-articular injections, Pediatric rheumatology, Pain management

## Abstract

**Background:**

Intra-articular corticosteroid injections (IACI) are routinely used by pediatric rheumatologists in the treatment of chronic arthritis. Frequently, topical anesthetics are used to control procedural pain, but their relative efficacy has not been reported. In this study, we evaluated the level of pain associated with different anesthetic methods, Numby® 900 Iontophoretic Drug Delivery System, or EMLA® cream, with or without subcutaneous buffered lidocaine (SQBL), during IACI of the knee in children with arthritis.

**Methods:**

We conducted a prospective study of patients, ages 4 to 21 years old, followed at three pediatric rheumatology centers who were undergoing IACI of a knee joint. Patients were randomized into two treatment groups: 1) topical anesthetic only (EMLA® or Numby® (E/N)), or 2) topical anesthetic (E/N) and SQBL. Pain was assessed at baseline, during topical anesthetic placement, and following the IACI (post-procedure). The Faces Pain Scale-Revised (FPS-R), the Face, Leg, Activity, Cry, Consolability (FLACC) behavioral scale and the parental global assessment (PGA) (0 = best experience, 10 = worst experience) were determined.

**Results:**

Sixty-three patients (44 females) with a median [IQR] age of 10.8 [IQR = (8.2–14.4)] years (range 4.7–20 years) with active knee arthritis were consented. FPS-R post-procedure (*P* = 0.03), FLACC (*P* = 0.02) and PGA (*P* = 0.01) scores were significantly lower in females treated with E/N plus SQBL compared to patients treated with E/N only. Females in the E/N only group had a significant worsening of their baseline pain (*p* < 0.0004) and a greater magnitude of change in their baseline FPS-R scores (*p* < 0.001) from the procedure compared to females in the E/N plus SQBL group who had no worsening of their baseline pain. No significant change in pain level or PGA score was found among males in either treatment group. Pain scores overall were similar to the oligoarthritis patients, a more homogeneous group of patients. Both EMLA® (*n* = 33) and Numby® (*n* = 29) were equally well tolerated with no significant difference in median FPS-R administration scores overall.

**Conclusion:**

Our results suggest that a topical anesthetic plus SQBL is more effective for injection pain control than topical anesthesia only. Further studies addressing pain and anxiety will help determine the optimal method of pain control for IACI.

## Background

Intra-articular corticosteroid injections (IACI) are one of the mainstays of treatment for patients with chronic arthritis and are part of the 2011 American College of Rheumatology recommendations for the treatment of juvenile idiopathic arthritis (JIA) [1–4]. IACI enables the physician to deliver localized treatment and may reduce the need for, or serve as an adjunct to, systemic medications. IACI may be used in patients with reactive arthritis, JIA, Lyme arthritis, and arthritis secondary to an underlying autoimmune disease such as systemic lupus erythematosus. Survey data show that 90 % of pediatric rheumatologists would recommend IACI as either initial therapy or after unsuccessful therapy with non-steroidal anti-inflammatory drugs. Furthermore both parents preference and a decision analysis identified IACI as the preferred initial management of knee monoarthritis in JIA [2, 4].

Despite the frequent use of IACI, little attention has been paid to the pain and distress that may be associated with the procedure, and there is no established standard regarding the type of anesthesia to use for IACI. Both biological (i.e.,: age, sex) and psychological factors contribute to the fear and perception of pain and pain scores [5]. Minimizing the pain, anxiety and stress associated with the procedure is important to improve patient cooperation and to avoid unsuccessful procedural attempts [6]. Children experience pain, stress, and anxiety when undergoing IACI and it is important to treat the procedural pain both to limit suffering and to prevent lasting changes in pain systems and pain responses that may result in anticipatory anxiety and a heightened pain response with future procedures [6]. Being able to minimize pain and calm the patient will make the procedure more satisfying for all involved while ideally reducing the time it takes to perform the procedure.

Local anesthesia for IACI may include topical preparations such as EMLA® cream (AstraZeneca, Inc) or LMX-4 (Ferndale Laboratories) [1, 3], lidocaine iontophoresis, ethyl chloride spray (Gebauer Co.) and subcutaneously administered lidocaine [3]. Numby® 900 Iontophoretic Drug Delivery System (Iomed, Inc., Salt Lake City, UT) delivers a lidocaine HCL 2 % and epinephrine 1:100,000 topical solution into the skin under the influence of electric current. Lidocaine iontophoresis has a greater depth of penetration (8.6 mm after a 20 min application) [7] over a shorter period of time than EMLA® (5 mm after a 60 min application) [8]. Although one study found EMLA® to be ineffective for reducing the pain of IACI in children with juvenile rheumatoid arthritis, it is routinely used in clinical practice [1].

To determine the methods of local and general anesthesia currently being used by pediatric rheumatologists, a survey was conducted in 2006 of all members of the Childhood Arthritis & Rheumatology Research Alliance (CARRA), a major research collaborative organization of pediatric rheumatologists in North America [3]. Fifty-nine percent of respondents reported using EMLA®, while lidocaine iontophoresis was used by 11.8 % of respondents [3]. Sixty-six percent of physicians believe that giving subcutaneous lidocaine actually contributes to procedural pain. Sub-cutaneous buffered lidocaine was rarely used. Conscious sedation (CS) or general anesthesia (GA) was used by 85 % of physicians, usually at the discretion of the anesthesiologist [3]. The agents most frequently used were midazolam intra-venously (39.5 %), gas (i.e.,: nitrous oxide) (30 %) and propofol (19 %) or fentanyl or vistanyl (19 %) [3]. Interestingly, only 30 % of physicians use gas for IACI even though studies have shown it to be safe and effective for JIA patients undergoing IACI [9, 10]. Ideally, successful use of a local anesthetic would reduce the number of patients treated with CS or GA minimizing risks such as respiratory depression.

The anesthetic effect of EMLA® versus lidocaine iontophoresis for IACI has not been studied, nor is it known whether the addition of subcutaneous buffered lidocaine (SQBL) is beneficial. Our objective in this open-label prospective, randomized trial was to determine the optimal method of delivering local anesthesia for IACI by comparing two treatment groups: Numby® 900 Iontophoretic Drug Delivery System or EMLA® cream with or without subcutaneous buffered lidocaine (E/N + SQBL) or E/N only.

## Method

### Patients

Patients between 4 and 21 years of age with JIA or Lyme arthritis with chronic, active arthritis of at least one knee and who were prescribed an IACI were eligible for participation in the study and enrolled between April 2006 and November 2008. Exclusion criteria included: 1) allergy to lidocaine or any ingredient used in Numby® or EMLA®, 2) use of an electrically sensitive support system (i.e., pacemakers), 3) damaged skin or signs of infection at the proposed injection site, 4) coagulopathy, or 5) inability to evaluate pain associated with the procedure.

This study was approved by the Institutional Review Boards of Hackensack University Medical Center, Hackensack, NJ, St. Barnabas Medical Center, Livingston, NJ and Connecticut Children’s Medical Center, Hartford, CT. Written informed consent from parents and written informed assent when child >9 years old were obtained.

### Measures

The Faces Pain Scale-Revised (FPS-R) is a self-report measure consisting of 6 gender-neutral faces depicting increasing intensity of pain that corresponds to a 0–10 metric pain scale (0 = no pain,10 = worst pain). Studies have demonstrated that the FPS-R correlates highly with other self-report pain scales and children ≥4 years of age are able to use the scale [11]. Although often used for younger or non-verbal patients, a Face, Leg, Activity, Cry, and Consolability (FLACC) score was obtained in all patients, regardless of age. [12]. It is an observational pain scale that is used for young or sedated children and supplements self-report pain scales such as the FPS-R [12]. The FLACC score rates the above 5 parameters on a scale of 0–2 points for a maximum score of 10 (worst pain) [12]. A parental global assessment (PGA) was recorded based on the parent’s perceptions of the patient’s overall procedure experience at the completion of the procedure (0 = best experience, 10 = worst experience). All three of these measures were endorsed as outcome measures for acute pain trials by the Pediatric IMMPACT group [13].

### Procedure

Patients were randomized into one of four groups. Group 1 had 2.5 g of EMLA® applied with occlusive dressing (Tegaderm®; 3 M, St. Paul, MN) for 60 min. Group 2 had 2.5 ml of the lidocaine hydrochloride 2 % and epinephrine 1:100,000 topical solution placed on the Numby® 900 electrode pad and then placed on the patient at the injection site for 20 min. The dispersive pad was placed over a major muscle group 10–15 cm away from the drug electrode. Numby® 900 was started at 2.0 mA for 20 min. Group 3 had EMLA® applied as above plus an injection of SQBL (9:1 2 % lidocaine to sodium bicarbonate). Group 4 had Numby® 900 applied as above plus an injection of SQBL.

A FPS-R score was obtained from the patient at 3 time points: 1) upon arrival to the office (FPS-R baseline), 2) once the topical anesthetic was removed, before the needle was inserted (FPS-R topical anesthetic) to reflect discomfort from the anesthetic and 3) at completion of the procedure (FPS-R post-procedure) to reflect procedure pain. A FLACC score and PGA were recorded.

### IACI procedure

Parents were present during the procedure. A child life specialist or experienced nurse was present to provide psychosocial support, distraction and to improve patients’ and parents’ understanding of the procedure. After the skin was sterilized with povidone-iodine and alcohol, a 21 gauge needle on a 3 ml syringe was inserted into the joint space through a medial approach. In the SQBL group, the syringe was filled with buffered lidocaine and the periarticular space was slowly infiltrated at physician discretion. The needle was then advanced into the joint space, syringe was then replaced with a sterile empty syringe and the synovial fluid was aspirated. With the needle remaining in place, the contents of a syringe containing 1 mg/kg (maximum 100 mg) of triamcinolone hexacetonide was injected.

### Statistical analysis

Continuous random variables were summarized as mean (standard deviation) or median (inter-quartile range) depending on whether or not the data followed the normal distribution. Categorical responses were presented as frequencies (percentages). With a two-tailed test with significance level of 5 % and a defined clinically significant difference of 1.5 standard deviations on the primary outcome, pain, and power of 80 %, we calculated that a sample size of 72 patients (18/group) was required. Because this target was not achieved, we aggregated the four treatment groups into two treatment groups a) patients who received EMLA® or Numby® only (E/N) and b) patients who received EMLA® or Numby® plus SQBL (E/N + SQBL). Continuous variables, age, disease duration, pain scores and global assessment were not normally distributed as indicated by the Shapiro-Wilk test. Hence, comparisons between any two groups were conducted using a Wilcoxon rank sum test. Comparisons of two groups with respect to categorical variables, gender, disease type, were performed using Chi-square test or Fisher Exact test as appropriate.

Subgroup analysis of pain scores was conducted in the cohort of oligoarthritis patients, the most common JIA subtype and a more homogeneous group of patients. Females reportedly have higher pain scores [5], so comparative analyses examined if sex effects were significant. Wilcoxon signed-rank test was used to compare the changes in FPS-R (post-procedure) - FPS-R (baseline). Further, the paired differences were compared across pain management methods using Wilcoxon rank sum test taking into account patients’ pain score at baseline. All statistical analyses were performed using SAS software version 9.2 (SAS Institute Inc., Cary, NC).

## Results

Seventy-seven patients were approached for the study and 63 (44 girls) (Table 1) agreed to participate. Reasons for refusal were preference for topical anesthesia (13 patients) or refusal to have a child-life therapist present (1 patient). The median age of study patients was 10.8[IQR = (8.2 to 14.4)] years (range 4.7–20 years), and over 80 % were Caucasian. There was no significant difference between treatment groups for disease duration or any demographic parameter (Table 1).

**Table 1:** Characteristics of arthritis patients receiving intra-articular corticosteroid injections

Characteristics	All	EMLA® or Numby®^b^	EMLA® or Numby® + Buffered Subcutaneous Lidocaine^b^
Total n (%)	63 (100)	28 (44.4)	35 (55.6)
Age^a^ (years)	10.8 (8.2 – 14.4)	11.5 (8.7 – 13.1)	10.5 (8.2 – 14.6)
Range (years)	4.7 – 20	4.7 – 17.9	6.1 – 20.0
Gender			
Male	19 (30.2)	9 (32.1)	10 (28.6)
Female	44 (69.8)	19 (67.9)	25 (71.4)
Disease Duration^a^ (years)	1.2 (0.2 – 4.8)	1.4 (0.11 – 4.7)	1.2 (0.2 –5.0)
Range (years)	0 – 15.6	0.0 – 9.6	0.02 – 15.6
Disease Type			
Oligoarthritis	49 (77.8)	24 (85.7)	25 (71.4)
Polyarthritis	7 (11.1)	2 (7.1)	5 (14.2)
Systemic arthritis	1 (1.6)	0 (0.0)	1 (2.9)
Enthesitis-Related arthritis	1 (1.6)	1 (3.6)	0 (0.0)
Psoriatic arthritis	2 (3.2)	1 (3.6)	1 (2.9)
Lyme arthritis	3 (4.8)	0 (0.0)	3 (8.6)

^a^Median [inter-quartile range (IQR) = 25^th^- 75^th^ percentile]. ^b^No significant difference between groups

In the E/N only group (*n* = 28), the median FPS-R baseline score was 0.0 [IQR = (0.0 to 2.0)] and the median FPS-R post-procedure score was higher at 3.0 [IQR = (2.0 to 6.0)]. In the E/N plus SQBL group (*n* = 35), both the median FPS-R baseline score and FPS-R post-procedure score were 2.0 [IQR = (0.0 to 4.0)] and 2.0 [IQR = (1.0 to 4.0)], respectively. A comparison between patients in the two treatment groups using the FPS-R post-procedure and FLACC scores did not reveal any significant differences. There was a significant difference in the PGA (*P* = 0.005).

Looking at all patients, there was no significant difference between treatment groups with regards to mild, moderate and severe pain. When examining the groups based on gender, the female patients in the E/N only group had significantly worse procedure pain (*P* = 0.03) with 21 % of patients having severe pain (FPS-R score 7.0–10.0) (Table 2) [14]. There was no significant difference between males.

**Table 2:** Pain severity of intra-articular corticosteroid injections between treatment groups using Faces Pain Scale-Revised post-procedure scores

Patients	Pain severity	EMLA® or Numby® (*n* = 28)	EMLA® or Numby® + Buffered Lidocaine (*n* = 35)	*P*-value
All (*n* = 63)	Mild	14 (50.0)	23 (65.7)	
	Moderate	10 (35.7)	11 (31.4)	0.2367
	Severe	4 (14.3)	1 (2.7)	
Male (*n* = 19)				
	Mild	7 (77.8)	7 (70.0)	
	Moderate	2 (22.2)	2 (20.0)	1.000
	Severe	0 (0.0)	1 (10.0)	
Female (*n* = 44)				
	Mild	7 (36.8)	16 (64.0)	
	Moderate	8 (42.1)	9 (36.0)	0.0260*
	Severe	4 (21.1)	0 (0.0)	

All data entries are counts (percentages). Pain scores: Mild: (0–3.9); Moderate (4.0 –6.9); Severe (7.0 –10.0). *P < 0.05 was considered statistically significant

Because it was determined that there was a significant interaction between gender and FPS-R (*P* = 0.02), FLACC (*P* = 0.04) and PGA (*P* = 0.005) scores, subsequent comparisons between treatment groups were performed within each gender (Table 2). Females in the E/N plus SQBL group had significantly lower FPS-R, FLACC and PGA post procedure scores. Overall, males (*n* = 19) in the two treatment groups did not have a significant difference in median pain or PGA scores. These findings held true even when the more homogenous group of oligoarticular JIA patients were compared (Table 3).

**Table 3:** Pain scores post-procedure in childhood arthritis by treatment group

	Male (*n* = 19)			Female (*n* = 44)		
	EMLA® or Numby® (*n* = 9)	EMLA® or Numby® + Subcutaneous Buffered Lidocaine (*n* = 10)	*P*-value	EMLA® or Numby® (*n* = 19)	EMLA® or Numby® + Subcutaneous Buffered Lidocaine (*n* = 25)	*P*-value
All patients (63)						
FPS-R (63)	2.0 (2.0–2.0)	1.5 (0.0–4.0)	0.64	5.0 (2.0–6.0)	2.0 (2.0–4.0)	0.03*
FLACC (56)	0.0 (0.0–1.0)	1.0 (0.0–2.0)	0.53	2.5 (0.0–6.0)	0.0 (0.0–3.0)	0.02*
PGA (61)	2.0 (1.0–2.0)	0.5 (0.0–2.0)	0.37	2.0 (1.0–5.0)	0.0 (0.0–2.0)	0.01*
Oligoarthritis patients (49)						
FPS-R (49)	2.0 (2.0–4.0)	1.0 (0.0–2.0)	0.25	6.0 (2.0–6.0)	2.0 (2.0–4.0)	0.06
FLACC (42)	1.0 (0.0–1.0)	1.0 (0.0–4.0)	0.74	3.5 (0.5–7.0)	0.0 (0.0–2.0)	0.01*
PGA (48)	2.0 (1.0–2.0)	1.5 (0.0–2.0)	0.54	2.0 (1.0–3.0)	0.0 (0.0–1.0)	0.01*

Pain scores are reported as Median (IQR: 25^th^-75^th^ percentiles); IQR = inter quartile range, FPS-R = Faces Pain Scale-Revised, FLACC = Faces, Leg, Affect, Cry, Consolability scale, PGA = parental global assessment. Two- sided Wilcoxon Rank Sum Test was used to compare pain scores between groups. *P < 0.05 was considered statistically significant

Nineteen patients (31 %) (13 F) had a prior IACI. The Friedman rank test revealed a significant interaction between gender and prior IACI with the FPS-R (*P* > 0.03), therefore, comparisons were performed within each gender. With the exception of the FLACC scores in males, those patients who had a prior IACI did not experience worse pain compared to patients who never had a prior IACI, independent of their gender (*P* > 0.05).

**Fig. 1:**
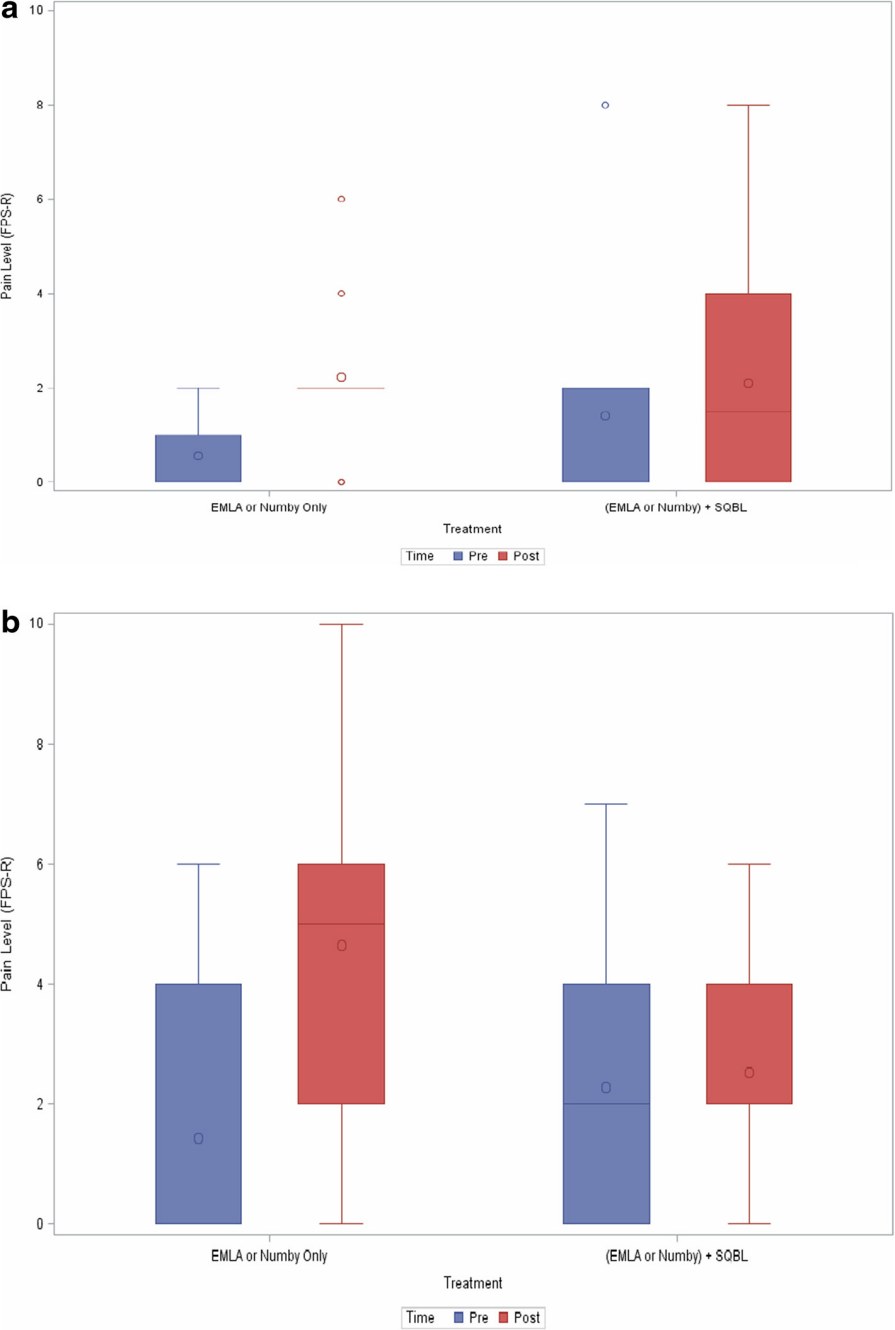
**a** Comparison of Faces Pain Scale-Revised (FPS-R) pre- and post-procedure scores in male patients (*n* = 19) during intra-articular corticosteroid injection. **b** Comparison of Faces Pain Scale-Revised (FPS-R) pre- and post-procedure scores in female patients (*n* = 44) during intra-articular corticosteroid injection

Table 4 compares the change in FPS-R scores from baseline to post-procedure between treatment groups within each gender. Females in the E/N group had a significant worsening (*p* = 0.0004) of their baseline pain (pain related to their active arthritis) by 2 points compared to the females in the E/N plus SQBL group that had no worsening of their baseline pain from the procedure (Table 4, Fig. 1). Within each topical anesthetic treatment group the change in FPS-R scores from baseline to post-procedure were examined, and females in the E/N only group had worsening of their baseline pain from the procedure (*p* = 0.001) (results not shown). No significant change in pain level was found among males either between or within treatment groups.

**Table 4:** Comparison of Faces Pain Scale-Revised (FPS-R) scores from IACI in childhood arthritis between treatment groups by gender

	Male (*n* = 19)		Female (*n* = 44)			
	EMLA® or Numby® Only (*n* = 9)	EMLA® or Numby® + Subcutaneous Buffered Lidocaine (*n* = 10)		EMLA® or Numby® Only (*n* = 19)	EMLA® or Numby® + Subcutaneous Buffered Lidocaine (*n* = 25)	
	Change in FPS-R score	Change in FPS-R score	*P*-value	Change in FPS-R score	Change in FPS-R score	*P*-value
All patients (*n* = 63)	1.0 (0.0–2.0)	0.5 (0.0–2.0)	0.61	2.0 (2.0–5.0)	0.0 (0.0–2.0)	0.0004*
Oligo-arthritis (*n* = 49)	2.0 (0.0–4.0)	0.0 (0.0–2.0)	0.16	2.0 (2.0–5.0)	0.0 (−1.0–1.0)	0.0006*

Pain scores are reported as Median (IQR: 25^th^-75^th^ percentiles); Change in FPS-R scores = FPS-R post-procedure – FPS-R baseline; Comparison of the changes in FPS-R scores between treatment groups were examined using Wilcoxon rank sum test. IACI = intra-articular corticosteroid injection. *P < 0.05 was considered statistically significant

Both EMLA® (*n* = 33) and Numby® (*n* = 29) were equally well tolerated with no significant difference in median FPS-R administration scores overall. Adverse events were minor. In the Numby® 900 group, they included: blanching (*n* = 17), redness (*n* = 13), and complaints of “tingling” (*n* = 14), “itching” (*n* = 7), “pain” (*n* = 6), and “burning” (*n* = 4). In the EMLA® group, they included blanching (*n* = 21), redness (*n* = 9), and complaints of “itching” (*n* = 1).

## Discussion

The results of our study suggest that a combination of E/N plus SQBL along with supportive staff to aid in relaxation techniques offers improvement in pain control compared to E/N alone for the IACI procedure. Over 40 % of clinicians feel that parent and patient anxiety were issues regarding the type of anesthesia to be used for IACI, and even if the IACI should be prescribed at all [2, 3]. Clinician’s also viewed insufficient nursing or medical support and insufficient means for patient sedation as obstacles to IACI [2]. Our study results will reassure clinicians, patients, and their families that pain from IACI is mild in most cases and may encourage clinicians to do more injections in the office with only local anesthesia, and not use conscious sedation, if support staff such as a child life therapist or a skilled nurse is available for support of the patient and parents.

Patients in the E/N plus SQBL group had higher baseline pain scores, however, there was no statistically significant difference between the two groups. Those patients with the higher baseline pain scores may have a lower threshold for pain or greater anxiety about the upcoming procedure.

Those patients not treated with SQBL during the IACI procedure had worsening of their baseline arthritis pain in contrast to the group that received the SQBL who had no worsening of their baseline pain (Table 4). Females who only received E/N tended to have higher median FPS-R post procedure scores and more severe procedure pain. These results are especially important since JIA overall has a female predominance, especially within the oligoarthritis sub-type.

More important than statistical significance is a minimal clinically important difference (MCID) in pain scores, in which the patient would find the procedure pain tolerable. Prior studies have shown a 2 point change in score out of 10 points (or 1 face) on the FPS to be clinically significant [15]. Our study has demonstrated a MCID in pain scores. Those patients that only received E/N had a worsening of their baseline pain as demonstrated by an increase in their score of 2 points (1 face) compared to the group that received SQBL who had no worsening of their pain from the procedure. Tsze, et al. defined mild pain as a score of 0–3.9, moderate pain as a score of 4–6.9 and severe pain as a score of 7–10 [14]. If we apply this definition to our cohort, the patients in the E/N plus SQBL group had minimal procedure pain, whereas, the FPS-R scores were moderate (5–6) in the E/N only group. Topical anesthetic plus SQBL resulted in excellent injection pain control as evidenced by low pain scores and a MCID in pain scores.

A contributing factor to our low pain scores may be our use of a child life therapist during the procedure. Child life therapists play an important role in helping patients cope with anxiety and procedure pain by reassuring and distracting them and by employing relaxation techniques. Studies have shown that developmentally appropriate behavioral and cognitive pain management strategies (i.e.,: deep breathing, reassurance) are often effective in pain control [16, 17]. Weintraub, et al. found that children with JIA undergoing IACI using self-administered nitrous oxide with a medical clown in attendance prior to and during the procedure had low visual analog scores of 1 [11].

Buffering the lidocaine, which is acidic (pH 3.5–7), with sodium bicarbonate and administering slowly is also an important step in reducing procedural pain as it reduces the burning sensation associated with its infiltration into skin (pH 7.35–7.45) [18]. A Cochrane review published in 2010 reported that both adult and pediatric patients preferred buffered lidocaine in both parallel and cross-over trials and no adverse events were reported [18].

Both topical anesthetics were well tolerated with no significant difference in FPS-R administration scores. Blanching was the most common side effect of both agents which is to be expected due to their vasoconstrictive properties. Our patients tolerated the electric current from the lidocaine iontophoresis which was an important finding since this modality has a faster onset of action and a greater depth of penetration than EMLA® cream. A study looking at determinants of success and failure of EMLA® found that while EMLA® has proven to be safe, it seems to offer less pain control, at least when applied for 60 min, compared to iontophoresis [19]. It is recommended that EMLA® be applied for a minimum of 90 min, preferably 120 min for intravenous cannulation [19]. Based on these findings, and reports by others regarding ease of use and tolerability, lidocaine iontophoresis may be the topical anesthetic of choice to use in a busy clinic setting [3, 6].

There were several limitations of our study including small sample size, open-label, and age of our study population. The number of patients needed to adequately power this study was not achieved. The median patient age in our study was 10.8 [IQR = (8.2 to 14.4)] years, so expanding the study to include a greater number of younger children would be useful to determine if our findings are applicable to children less than 5 years of age. This study was not blinded and therefore the patient may have a biased response to their treatment based on the physician’s or nurse’s attitudes about the anesthetic used. Normal synovium may be up to 45 mm thick, but inflamed synovium can be significantly thickened [20]. This can contribute to a more painful procedure, and ultrasound was not used to measure synovial thickness or as an injection guide.

## Conclusion

IACI are routinely used by pediatric rheumatologists and since patients frequently need repeat injections, it is important to identify strategies that minimize pain to better ensure patient cooperation and compliance with the current and future procedures. Our results suggest that the addition of subcutaneous buffered lidocaine to a topical anesthetic is an improvement over topical agents alone, especially in females. Future studies addressing pain and anxiety around IACI are needed to establish the best practices for performing IACI.

## Abbreviations

IACI: Intra-articular corticosteroid injection
EMLA: Eutectic mixture of local anesthetics
E/N: EMLA® or Numby®
SQBL: Subcutaneous buffered lidocaine
JIA: Juvenile idiopathic arthritis
FPS-R: Faces pain scale - revised
FLACC: Face, legs, arms, cry, consolability
PGA: Parental global assessment
CARRA: Childhood Arthritis & Rheumatology Research Alliance
SD: Standard deviation
IQR: Inter-quartile range
MCID: Minimal clinically important difference
